# Association Between Food Choices Motivators and Physical Activity in Body Image (dis)Satisfaction in Portuguese Adolescents

**DOI:** 10.3389/fpubh.2021.651228

**Published:** 2021-06-04

**Authors:** Sara Simões Dias, Marlene Lages, Roberta Frontini, Luís Luís, Maria dos Anjos Dixe, Pedro Sousa

**Affiliations:** ^1^ciTechCare - Center for Innovative Care and Health Technology, Polytechnic of Leiria, Leiria, Portugal; ^2^EpiDoC Unit, Chronic Diseases Research Center (CEDOC), NOVA Medical School, Universidade Nova de Lisboa, Lisboa, Portugal; ^3^CIEQV - Life Quality Research Centre, Polytechnic of Leiria, Leiria, Portugal; ^4^Health Sciences Research Unit: Nursing, Nursing School of Coimbra, Coimbra, Portugal

**Keywords:** body image, physical activity, sedentary behaviour, adolescents, public health, nutrition

## Abstract

Concerns about weight and body image are common among adolescents since they are particularly vulnerable to body-image dissatisfaction due to the normal physiological, social, and psychological changes they are going through. This study aims to analyse the relationship between food choice motivations and physical activity in body-image perception among adolescents. Twelve to sixteen years old adolescents were recruited from three school districts. The Portuguese version of the Food Choices Questionnaire (FCQ) was used to assess food choice motivators, and the Quantification de l'Activité Physique en Altitude Chez les Enfants was used to assess physical activity and to calculate daily energy expenditure (DEE). Body image perception was measured using Collins' sequence of seven silhouettes. Body image (dis)satisfaction was estimated by the present body shape minus the desired body shape. ANOVA and Kruskal-Wallis tests were performed to compare groups, and the *post-hoc* Bonferroni test was used to compare target groups. A multinominal logistic regression was performed to analyse the association between gender, age, hours of sport's competition, FCQ, and body dissatisfaction. All analyses were performed in IBM SPSS Statistics 26.0. The sample comprised 286 adolescents (51.4% females). Means of FCQ categories varied between 0.33 and 0.97 (range: −2 to 2). Regarding the categories of FCQ, statistically significant differences were found in the category of body satisfaction and weight control among the three groups (*p* = 0.004). A preventive effect was found of choosing food regarding body satisfaction and weight control, on body-image dissatisfaction.

## Introduction

Preventing overweight, obesity, and eating disorders in adolescents are issues of utmost importance. According to the World Health Organization, it is estimated that over 340 million children and adolescents aged 5–19 years are classified as overweight or obese (1). In Portugal, overweight, including obesity, is also high in adolescents, according to data from the National Food, Nutrition and Physical Activity Survey, 2015–2016, 23.6% of adolescents have pre-obesity and 8.7% have obesity (2). The current sedentary lifestyle and hypercaloric food patterns are the main causes of overweight and this, in association with the adoption of thinner body models, has dictated a high prevalence of body image dissatisfaction. Besides, the adolescence phase is considered crucial in the development of the body image whereby poor development of this construct may increase imbalances in the perception of the body image. Negative body image has been traditionally associated with adolescent disordered eating and obesity (3, 4). Studies have shown that a negative body image perception, which includes body dissatisfaction, is associated with poorer psychological and physical health, disordered eating (5) and overweight development in the future life of the adolescents (6, 7).

Body image, a multidimensional construct central to emotional well-being in which the attitudinal component is satisfaction with body size (8), is a strong determinant of nutritional habits and weight management practises among adolescents (9). The assessment of body image perception permits the quantification of a significant dimension of body image, namely, the satisfaction or dissatisfaction with the body (9). Body image (dis)satisfaction is usually measured as the difference between the perceived and the ideal body figure. Dissatisfaction with body image is a common concern between both genders, peaking during early adolescence (10). Overall, several factors may contribute to body image dissatisfaction in individuals of different age groups, such as biological, psychological, and sociocultural factors (11). In addition, body image dissatisfaction is strongly related to poor nutritional and physical activity (PA) habits and with unsafe weight management practises in adolescents (12). Adolescents who are underweight or have normal weight can sometimes see themselves as overweight, therefore increasing the risk of unhealthy weight management behaviours and, consequently, eating disorders (13). Contrary to this are the adolescents who are overweight and that often do not perceive themselves as such, decreasing the probability to adhere to healthy weight control practises, as PA engagement and healthy eating (14). As shown in a study conducted by Sánchez-Miguel et al. (15) physical activity is negatively related to the perception of body image.

Since overestimation and underestimation of weight are associated with a larger risk in adolescents, it becomes essential to understand the relations between body image and health behaviours to avoid conducts that compromised the health status (16, 17). Presently, data referring to the relation between body image perception, food choices and PA in adolescents is still scarce and remains unclear. In order to provide effective health interventions, it is essential to identify the aetiology of body dissatisfaction in Portuguese adolescents. Also, given the critical role of body image satisfaction in shaping adolescents' behaviours, it is crucial to thoroughly explore and comprehend its role in this development group. Such data are needed to accurately design and monitor health education programs, bringing an improvement in nutrition, PA counselling and weight management strategies amongst adolescents' populations. The current study, therefore, aims to determine the relationship between body image perception, food choices and PA among Portuguese adolescents.

## Methods

### Study Design and Participants

This is a population-based cross-sectional study conducted on adolescents aged between 12 and 16 years old, who attended three different middle schools. Data collection took place in Portugal during the school year, from October 2018 to March 2019. The application of the questionnaires was authorised by the Ministry of Education (registration number 0254300004) and approved by the National Data Protection Commission (n.° 11465/2017, October 12). The assessment protocol was completed in person by the child/adolescent and their parents in a consultation office provided for the purpose, after obtaining the written informed consent form from the parents and the adolescents older than 13 years, and the informal verbal assent from younger children. The complete study protocol is available for review (18).

### Data Collection

#### Sociodemographic Data

Information was collected on sociodemographic data, namely, age, gender, number of household members, and residential area of the adolescents that were participating in the study.

#### Food Choices Questionnaire (FCQ)

The Portuguese version of the FCQ was used to evaluate the influence of food choice motivations. This questionnaire consists of 26 questions, the answer to which is given on a scale ranging from totally agree to totally disagree. The FCQ is structured in five factors: body satisfaction and weight control; ethical concerns; convenience; sensitive qualities; and humour. For each factor, the average that varies between −2 and 2 is calculated, and the greater the result, the greater the individual's food options (19).

#### Body Image Perception

Collin's sequence of seven female or male silhouettes were used to measure body image perception (20). Each image corresponds to an increase in shape, from very thin (silhouette 1) to obese (silhouette 7). The adolescents were asked to select an image that best represents their actual body size corresponding to the perception of their current body shape, and the silhouette that represented their ideal body size. The discrepancy between the “real” body image and the “ideal” body image is used as an indicator of (dis)satisfaction with body image. Negative values indicate the desire to gain weight, positive values indicate the desire to lose weight and a value equal to zero indicated satisfaction with their body image (21).

#### Quantification de l'Activité Physique en Altitude Chez les Enfants (QAPACE)

Physical activity was assessed using the QAPACE, a self-administered questionnaire designed and validated for a range of ages between 8 and 16 years old (22). This questionnaire aims to quantify the levels of PA covering all the possible activities conducted by youth during the school and vacation periods. The questionnaire is composed by closed answers, and divides activities into daily activities, school activities (curricular and extracurricular), and extra-school activities (complementary, religious, domestic, holidays, competitive sports, and transportation). Thus, it can be used to measure daily energy expenditure (DEE) over the past year, during school period (sp) and vacation period (vp). The DEE was calculated according to the formula below of the original questionnaire (22), along with the information of the PA compendium (23).

$$DEE\;=\;\sum_{i\;=\;1}^{i\;=\;13}\left(((\left(f_{sp}\left(i\right).d_{sp}\left(i\right).280\right)+\left(f_{vp}\left(i\right).d_{vp}\left(i\right).85\right))/365)m(i)\right)$$

For each possible activity (*i* = 1–13), f(i) corresponds to its daily frequency, d(i) to its mean duration and m(i) to its intensity according to the compendium of physical activities (22). Sedentary activities were defined as energy expenditure of <1.5 MET (metabolic equivalents of task), excluding sleeping.

From the data collected with QAPACE, a new variable was built that measures the number of hours of competition sport practised by the adolescents.

## Statistical Analysis

Data were analysed using IBM SPSS software (version 26.0) and subjected to descriptive statistical analysis techniques (mean), dispersion and variability measures (standard deviation). Parametric tests, namely, ANOVA was computed to compare means between groups when the variables had a normal distribution and homogeneity of variances. Pearson chi-square test was used to assess differences between groups. Analysis of variance with *post-hoc* Bonferroni multiple comparison tests was used to compare target groups. Non-parametric tests (Kruskal-Wallis) were used for variables that did not meet the requirements of normal distribution and homogeneity of variances. Multinomial logistic regression was used to examine the relationship between gender, age, hours of sport's competition and categories of food choice motivators in body dissatisfaction. The referent group consisted of participants who reported that were satisfied with their image. Odds ratios, 95% confidence intervals, and *p*-values are presented for the multinomial model. Statistical significance was set at *p* <0.05.

## Results

The final sample comprises 280 adolescent respondents aged 12–16 years old, with more females (51.4%) than males (48.6%). The adolescents were divided into three groups, according to their degree of satisfaction or dissatisfaction with their body image, namely, satisfied, dissatisfied because they want to lose weight and dissatisfied because they want to gain weight. Of the total, only 36.0% of the adolescents were satisfied with their body image, 17.8% of the adolescents were dissatisfied because they want to gain weight, and the remain 46.2% were dissatisfied because they wanted to lose weight. Table 1 indicates the descriptive statistics according to the groups in the study.

**Table 1:** Descriptive statistics of students by level of (dis)satisfaction with body image perception.

	**Dissatisfied (want to gain weight)**	**Satisfied**	**Dissatisfied (want to lose weight)**	***p*-value**
Age $\bar{x\;}\pm$ s.d.)	12.33 ± 0.77	12.29 ± 0.73	12.35 ± 1.04	0.264^†^
Gender [*n* (%)]				
Female	24 (48.0%)	49 (50.0%)	71 (53.8%)	0.737^Ψ^
Male	26 (52.0%)	49 (50.0%)	61 (46.2%)	
Sport competition [($\bar{x\;}\pm$ s.d.) hours]	70.59 ± 143.63	124.51 ± 201.70	76.82 ± 167.20	0.076^†^
Female	38.13 ± 76.23	98.27 ± 178.98	55.77 ± 113.08	0.125^†^
Male	103.27 ± 183.29	163.47 ± 224.84	101.31 ± 212.09	0.269^†^
No. of household members ($\bar{x\;}\pm$ s.d.)	3.95 ± 0.72	3.81 ± 0.67	4.07 ± 1.10	0.370^†^
Residence area [*n* (%)]				
Rural area	5 (12.8%)	6 (7.1%)	12 (11.5%)	0.829^Ψ^
Urban area	22 (56.4%)	52 (61.9%)	59 (56.7%)	
Suburban area	12 (30.8%)	26 (31.0%)	33 (31.7%)	

† *ANOVA test*.

Ψ *Chi-square test*.

Concerning the five categories of the FCQ, statistically significant differences were only found in the category of “body satisfaction and weight control” among the three groups (Table 2). One-way ANOVA showed that there is an effect of the group on body satisfaction and weight control (*p* = 0.04). After the analysis according to gender, it was found that the statically significant difference only remained in the female group (*p* = 0.014). Regarding the category of the FCQ “body satisfaction and weight control,” Bonferroni's *post-hoc* showed significant differences between the group satisfied with their body image and the group dissatisfied because they want to lose weight (*p* = 0.007).

**Table 2:** Mean values of the categories of the Food Choices Questionnaire according to the levels of the body (dis)satisfaction.

	**Dissatisfied (want to gain weight) (*n* = 51)**	**Satisfied (*n* = 103)**	**Dissatisfied (want to lose weight) (*n* = 132)**	***p*-value**
Body satisfaction and weight control	0.65 ± 0.75	0.65 ± 0.78	0.94 ± 0.64	0.040^*^
Ethical concerns	0.47 ± 0.78	0.48 ± 0.82	0.49 ± 0.83	0.986
Convenience	0.33 ± 0.71	0.34 ± 0.90	0.41 ± 0.81	0.734
Sensitive quality	0.97 ± 0.73	0.96 ± 0.72	0.92 ± 0.82	0.919
Humour	0.85 ± 0.70	0.87 ± 0.76	0.91 ± 0.75	0.844
Total	0.70 ± 0.47	0.69 ± 0.54	0.79 ± 0.52	0.330
**Male**	**(*****n*** **=** **26)**	**(*****n*** **=** **49)**	**(*****n*** **=** **61)**	
Body satisfaction and weight control	0.63 ± 0.65	0.69 ± 0.74	0.90 ± 0.63	0.145
Ethical concerns	0.55 ± 0.75	0.60 ± 0.82	0.55 ± 0.81	0.947
Convenience	0.41 ± 0.70	0.49 ± 0.86	0.40 ± 0.82	0.508
Sensitive quality	0.98 ± 0.66	1.00 ± 0.64	0.92 ± 0.79	0.848
Humour	0.99 ± 0.66	0.75 ± 0.84	0.94 ± 0.73	0.323
Total	0.73 ± 0.43	0.72 ± 0.52	0.77 ± 0.50	0.851
**Female**	**(*****n*** **=** **24)**	**(*****n*** **=** **49)**	**(*****n*** **=** **71)**	
Body satisfaction and weight control	0.68 ± 0.86	0.56 ± 0.83	0.97 ± 0.66	0.014^*^
Ethical concerns	0.38 ± 0.84	0.29 ± 0.79	0.44 ± 0.84	0.624
Convenience	0.31 ± 0.70	0.14 ± 0.89	0.50 ± 0.78	0.061
Sensitive quality	0.96 ± 0.84	0.85 ± 0.77	0.93 ± 0.86	0.834
Humour	0.74 ± 0.74	0.92 ± 0.67	0.89 ± 0.78	0.583
Total	0.68 ± 0.51	0.61 ± 0.53	0.81 ± 0.53	0.169

*Data are presented as mean ± standard deviation*.

* *p <0.05*.

Table 3 indicates the mean weekly hours that the adolescents spent on different sedentary and sports activities during school and vacation periods. The mean time spent in sedentary activities is higher in both groups that are dissatisfied with their body image, however, no statistically significant difference was found (*p* > 0.05). Regarding the average time spent weekly on sports activities, this value was higher in the group dissatisfied with their body image (wants to gain weight), both during school and on vacation, but no statistically significant differences were found (*p* > 0.05).

**Table 3:** Descriptive statistics of the duration of time spent by the students on sedentary and sports activities.

	**Dissatisfied (want to gain weight)**	**Satisfied**	**dissatisfied (want to lose weight)**	***p*-value**
**Sedentary and sports activities during the school period**				
Watching TV (hours/week)	5.31 ± 2.37	4.43 ± 3.07	5.68 ± 3.57	0.129
Videogames (hours/week)	6.63 ± 4.49	5.86 ± 4.36	6.88 ± 4.71	0.506
Listening to music (hours/week)	5.34 ± 4.49	4.45 ± 3.36	5.47 ± 3.48	0.352
Reading (hours/week)	3.09 ± 2.86	2.13 ± 1.67	3.11 ± 2.64	0.176
Sports (hours/week)	5.23 ± 3.23	4.86 ± 3.29	3.65 ± 2.43	0.107
**Sedentary and sports activities during the vacation period**				
Watching TV (hours/week)	6.61 ± 4.15	5.35 ± 3.87	6.84 ± 4.04	0.162
Videogames (hours/week)	8.09 ± 4.73	7.10 ± 4.16	8.43 ± 4.81	0.345
Listening to music (hours/week)	5.73 ± 4.15	5.65 ± 4.14	6.99 ± 4.69	0.269
Reading (hours/week)	3.46 ± 2.42	3.05 ± 2.71	3.49 ± 2.34	0.743
Sports (hours/week)	7.00 ± 4.09	5.77 ± 3.38	5.52 ± 4.36	0.495
**Male**				
**Sedentary and sports activities during the school period**				
Watching TV (hours/week)	5.98 ± 2.64	4.13 ± 2.79	5.11 ± 3.32	0.185
Videogames (hours/week)	6.25 ± 3.95	5.97 ± 4.33	8.15 ± 4.80	0.165
Listening to music (hours/week)	3.72 ± 3.06	4.29 ± 3.10	6.38 ± 3.99	0.096
Reading (hours/week)	3.40 ± 3.31	1.75 ± 1.45	2.23 ± 1.74	0.273
Sports (hours/week)	7.21 ± 3.28	5.51 ± 3.14	4.41 ± 2.78	0.150
**Sedentary and sports activities during the vacation period**				
Watching TV (hours/week)	7.77 ± 4.55	5.25 ± 4.06	7.72 ± 4.44	0.103
Videogames (hours/week)	8.19 ± 4.22	7.50 ± 4.35	10.38 ± 4.46	0.055
Listening to music (hours/week)	5.28 ± 4.15	6.31 ± 4.13	8.19 ± 4.98	0.238
Reading (hours/week)	3.88 ± 2.43	2.61 ± 2.06	2.17 ± 1.30	0.200
Sports (hours/week)	7.13 ± 4.38	5.63 ± 3.23	8.08 ± 5.43	0.368
**Female**				
**Sedentary and sports activities during the school period**				
Watching TV (hours/week)	4.28 ± 1.44	4.63 ± 3.37	6.10 ± 3.80	0.173
Videogames (hours/week)	7.25 ± 5.46	5.79 ± 4.68	5.80 ± 4.42	0.685
Listening to music (hours/week)	6.10 ± 4.91	4.70 ± 3.59	4.97 ± 3.11	0.455
Reading (hours/week)	2.92 ± 2.78	2.42 ± 1.84	3.60 ± 2.94	0.332
Sports (hours/week)	3.50 ± 2.09	4.48 ± 3.37	3.21 ± 2.14	0.281
**Sedentary and sports activities during the vacation period**				
Watching TV (hours/week)	5.25 ± 3.31	5.46 ± 3.74	6.27 ± 3.71	0.614
Videogames (hours/week)	7.95 ± 5.56	6.39 ± 4.02	6.79 ± 4.52	0.692
Listening to music (hours/week)	6.00 ± 4.27	5.37 ± 4.13	6.41 ± 4.50	0.670
Reading (hours/week)	3.08 ± 2.49	3.56 ± 3.24	3.93 ± 2.46	0.715
Sports (hours/week)	6.69 ± 3.84	5.90 ± 3.63	4.30 ± 3.22	0.258

*Data are presented as mean ± standard deviation*.

The multinomial logistic models (Table 4) show that hours of sport's competition, body satisfaction, and weight control were the only significant factors to explain body (dis)satisfaction. Those with higher values of body satisfaction and weight control and those with more hours of sport's competition are more likely to want to lose weight. This is relevant in all participants and is also relevant when we analyse male adolescents.

**Table 4:** Multinomial logistic regression models predicting body image (dis)satisfaction.

	**Total (*****n*** **=** **268)**		**Female (*****n*** **=** **140)**		**Male (*****n*** **=** **128)**	
**Variable**	**OR (95% CI)**	***p*-value**	**OR (95% CI)**	***p*-value**	**OR (95% CI)**	***p*-value**
Y = dissatisfaction (want to gain weight)						
Male	1.14 (0.56–2.30)	0.722	-	-	-	-
Age	0.93 (0.60–1.45)	0.745	0.82 (0.42–1.61)	0.558	0.99 (0.54–1.83)	0.986
Sport competition	1.00 (0.99–1.00)	0.121	1.00 (0.99–1.00)	0.132	1.00 (0.99–1.00)	0.429
Body satisfaction and weight control	1.05 (0.65–1.71)	0.832	1.16 (0.60–2.24)	0.653	0.91 (0.44–1.89)	0.797
Y = dissatisfaction (want to lose weight)						
Male	0.94 (0.54–1.65)	0.837	-	-	-	-
Age	1.26 (0.92–1.72)	0.152	1.37 (0.89–2.11)	0.153	1.13 (0.69–1.85)	0.627
Sport competition	1.00 (0.99–0.99)	0.010	1.00 (0.99–1.01)	0.319	1.00 (0.99–1.00)	0.020
Body satisfaction and weight control	1.89 (1.27–2.80)	0.002	1.91 (1.14–3.19)	0.014	1.86 (1.00–3.47)	0.050

The descriptive statistics of DEE are presented in Table 5, however, the sample size has been greatly reduced when this variable was calculated since most participants had not completed the QAPACE questionnaire or had an incomplete questionnaire. The group that was dissatisfied with their body image because they wanted to gain weight have the highest DEE mean value when compared with the other groups; however, this difference was not statically significant (Table 5).

**Table 5:** Descriptive statistics of daily energy expenditure of all the activities described in the *Quantification de l'Activite Physique en Altitude Chez les Enfants* questionnaire.

	**Dissatisfied (want to gain weight) (*n* = 8)**	**Satisfied(*n* = 20)**	**Dissatisfied (want to lose weight) (*n* = 17)**	***p***
DEE (Kcal) ($\bar{x\;}\pm$ s.d.)	1177.43 ± 871.63	892.47 ± 355.27	853.18 ± 267.93	0.709^‡^
Md (Q1–Q3)	879.03 (733.93–1444.03)	883.30 (626.90–1190.55)	786.00 (645.75–1175.25)	
Minimum DEE (Kcal)	451.20	387.40	507.40	
Maximum DEE (Kcal)	3036.80	1828.50	1356.00	
**Male**	**(*****n*** **=** **5)**	**(*****n*** **=** **6)**	**(*****n*** **=** **4)**	0.775
DEE (Kcal) ($\bar{x\;}\pm$ s.d.)	1204.82 ± 1039.78	727.73 ± 276.74	785.80 ± 199.42	
Md (Q1–Q3)	824.80 (614.35–1985.30)	630.70 (522.88–970.83)	835.75 (577.05–944.60)	
Minimum DEE (Kcal)	451.20	460.70	507.40	
Maximum DEE (Kcal)	3036.80	1209.40	964.30	
**Female**	**(*****n*** **=** **3)**	**(*****n*** **=** **13)**	**(*****n*** **=** **13)**	0.357
DEE (Kcal) ($\bar{x\;}\pm$ s.d.)	1131.77 ± 415.38	1001.29 ± 355.50	873.91 ± 289.47	
Md (Q1–Q3)	1125.80 (719.40–1125.80)	920.40 (782.95–1229.80)	754.30 (645.75–1235.05)	
Minimum DEE (Kcal)	719.40	387.40	597.70	
Maximum DEE (Kcal)	1125.80	1828.50	1356.00	

*DEE, daily energy expenditure. Md, median. s.d., standard deviation*.

‡ *Kruskal-Wallis test*.

Also, there were no differences in median DEE value between the three categories, neither in the sociodemographic data (sex and area of residence), with no statistically significant results found (*p* > 0.05).

## Discussion

The main aim of the present study was to analyse the association between body image dis(satisfaction) and two factors, namely, food choices motivators and physical activity levels. An additional objective was to calculate the DEE and analyse the relationship with the categories of body image dis(satisfaction). The results of the present study confirm a high prevalence of body image dissatisfaction in adolescents. In this study, we found that the prevalence of body image dissatisfaction was 64% in total. Similar results were observed in other studies conducted in Portuguese adolescents (24, 25) and Brazilian adolescents (26, 27). The similitude between the prevalences can be explained by social and cultural similarities between Portugal and Brazil.

Although Collins' sequence of silhouettes scales (20) is a cost-effective assessment tool to determine body (dis)satisfaction since it is easy and quick to apply, it can have possible methodological limitations (28). The most significant point concerns the arrangement of the figures within the scale. All figural drawings are presented on a single piece of paper and are arranged in an ascending sequence of sizes from left thin to right obese. This arrangement can cause a reporting bias towards thinner figures (29, 30).

Regarding differences between genders, several studies indicate that body dissatisfaction is more common amongst girls and women (25, 31, 32). However, while in the past this concern was more associated with women, nowadays the percentage of body image dissatisfaction is similar in both genders (24) but with different reasons regarding the dissatisfaction. Data shows that, in comparison with boys, more girls want to lose weight while more boys desire to gain weight (24, 25), which is consistent with the results found in this study. One possible explanation is that girls are more susceptible and influenced to pressure from the standards that are defined by society for body image (33). Besides, girls might be susceptible and affected by the order of the silhouettes in comparison with boys (34), but further research is needed on this topic.

Several factors can influence weight control practises, being the self-perception of body shape an important one (35). In this particular study, we assessed body image (dis)satisfaction, food choices and PA among adolescents in Portugal. Body image is an important target of intervention to improve health in adolescents, especially since it has been documented that worrying about body image is particularly acute during puberty (36). It has been previously reported that adolescents that are overweight and perceived themselves as such may actively try to lose weight (37), while the ones who do not perceive themselves as overweight can be less driven to take the steps to lose weight (38). Body image dissatisfaction in overweight individuals may lead to healthy weight management behaviours, including increased intake of healthy foods such as fruits and vegetables and regular PA (12, 39). On the other side, a poor body image may act as a barrier to practise physical activity leading adolescents with body image dissatisfaction to spent more time in sedentary activities such as watching television and playing video games (40, 41). According to Coelho et al. (24), the time spent watching television is a risk factor associated with body image dissatisfaction while sports activity has been identified as a variable with a preventive factor for image dissatisfaction, as reported in a cross-sectional study with Portuguese adolescents, where higher levels of PA were associated with a protective effect on body dissatisfaction (42). Physical activity was also associated with a more positive body image in two meta-analyses studies (26, 27). While PA has been reported in the literature as associated with body satisfaction, results from our study showed no differences between adolescents with body image satisfaction or dissatisfaction, regarding the time spent in sports. In line with these findings, Gonzaga et al. (11) reported no difference in body dissatisfaction among adolescents who practised and did not practise PA (11), while other studies have shown that physically active adolescents reported more dissatisfaction when compared to sedentary or low-activity participants (24, 43). These variances in results might be explained by the different methods select to assess PA and body (dis)satisfaction. In addition, the differences may also result from the low number of QAPACE questionnaires completed by the population in our study, and this subgroup may not represent the total sample. In the current study, the average time spent on sedentary activities by adolescents was similar to the time allocated to sports activities. However, a study conducted by Jalali-Farahani et al. (44) reported a lower time spent in sports activities.

Comprehend how body (dis)satisfaction affects food preferences and the overall adolescent diet is a key issue to develop strategies aimed at influencing dietary behaviour. Concerns regarding body image and weight control seem to have a strong effect on food choice for general populations. The interpretation of these data must consider the possible recall bias underlying the use of questionnaires, as is the case with the FCQ. The scale used in the FCQ has only five categories, which may not allow participants with a relatively strong opinion to voice a subtle position being required to choose a most extreme answer.

Overall, the current results of this study emphasise the relevance of body (dis)satisfaction on adolescent food choices. The present work has the advantage of studying dissatisfaction with body image from two different perspectives: dissatisfaction due to deficit (desire to gain weight) and excess (desire to lose weight). Several studies analyse only the satisfaction and dissatisfaction dimensions, which limits the comparability of the results presented.

However, this study presents some limitations, including (i) incomplete filling of the questionnaires by the adolescents and missing data that would allow the calculation of the DEE values in a larger sample of the study; and (ii) the cross-sectional design, which limits the ability to determine a causal relationship between food choice motivators, PA and body image perception. Longitudinal and prospective research is required to determine whether low levels of PA or sedentary behaviours can result in body image dissatisfaction or vice versa.

In future research, it would be equally important to better understand the eating patterns of these adolescents, through the use of questionnaires that allow collecting more detailed information on this component. There is a need for additional research exploring the food choice motives in conjunction with research investigating dietary intake to establish if the intention translates into practise. Besides, it would also be important to explore more the potential association between misperception of body size and body image satisfaction, as well as it will also be important to take into account other variables that may affect this construct, such as BMI and gender.

The data hereby presented could be beneficial to healthcare professionals and other practitioners in targeting educational messages for adolescents' specific eating patterns and to community planners in encouraging the availability of healthy food and promotion of the PA.

## Conclusions

The present study highlights the need to do more research in this area to better understand the associations between body image perception and lifestyle choices. In the meantime, new specific intervention strategies should be considered to increased awareness regarding food choices and PA to prevent future body-image disorders and decrease the prevalence of obesity, bringing clinically beneficial health outcomes. In the future, it should be developed a new social environment fostering the perception and acceptance of the real body image and public health should focus more on correct awareness of body size, preventing pathologies associated with body distortions. Taking into consideration the potentially positive role of PA and the negative role of sedentary activities on middle school students, governments and community healthcare professionals should incorporate these contributing factors into future policies and interventions targeted to adolescents.

## Data Availability Statement

The raw data supporting the conclusions of this article will be made available by the authors upon request, without undue reservation.

## Ethics Statement

Ethical review and approval was not required for the study on human participants in accordance with the local legislation and institutional requirements. Written informed consent to participate in this study was provided by the participants' legal guardian/next of kin.

## Author Contributions

SD, RF, LL, MD, and PS contributed to the conception and design of the study. PS, RF, SD, and ML organised the database. SD, ML, and RF performed the statistical analysis. SD and ML wrote the first draught of the manuscript. All authors have been involved in revising the manuscript critically for intellectual content. All authors read and approved the final manuscript.

## Conflict of Interest

The authors declare that the research was conducted in the absence of any commercial or financial relationships that could be construed as a potential conflict of interest.
